# Epigenetic Regulation of Apoptosis and Cell Cycle in Osteosarcoma

**DOI:** 10.1155/2011/679457

**Published:** 2010-12-27

**Authors:** Krithi Rao-Bindal, Eugenie S. Kleinerman

**Affiliations:** ^1^Department of Pediatrics, The University of Texas MD Anderson Cancer Center, Houston, TX 77030, USA; ^2^Division of Pediatrics, Unit 87, The University of Texas MD Anderson Cancer Center, 1515 Holcombe Boulevard, Houston, TX 77030, USA

## Abstract

The role of genetic mutations in the development of osteosarcoma, such as alterations in p53 and Rb, is well understood. However, the significance of epigenetic mechanisms in the progression of osteosarcoma remains unclear and is increasingly being investigated. Recent evidence suggests that epigenetic alterations such as methylation and histone modifications of genes involved in cell cycle regulation and apoptosis may contribute to the pathogenesis of this tumor. Importantly, understanding the molecular mechanisms of regulation of these pathways may give insight into novel therapeutic strategies for patients with osteosarcoma. This paper serves to summarize the described epigenetic mechanisms in the tumorigenesis of osteosarcoma, specifically those pertaining to apoptosis and cell cycle regulation.

## 1. Introduction

Osteosarcoma is the most common primary malignant tumor of the bone in children and adolescents. As a result of recent advances in chemotherapy, long-term survival rates for osteosarcoma patients with no detectable metastases at diagnosis have improved dramatically. However, for patients that present with metastasis or have disease recurrence, the long-term survival rate is less than 20% [1–3]. Therefore, there is an ongoing need to understand the biology of osteosarcoma progression and metastasis in order to identify new therapeutic approaches. 

Numerous studies have investigated the pathogenesis of osteosarcoma. This tumor has generally been associated with alterations in genes involved in cell cycle regulation and apoptosis. Most notably, the p53 and retinoblastoma protein (Rb) pathways have been shown to play a role in the progression of osteosarcoma [4–7]. However, much of the focus has been on understanding point mutations or deletions, disregarding any potential role of epigenetic mechanisms in the inactivation of these and other important pathways. 

Epigenetics involves changes in the activation of genes without altering the basic structure of DNA. This includes but is not limited to CpG island methylation within gene promoter regions and acetylation, deacetylation, and/or methylation of histone proteins [8, 9]. Epigenetic regulation has been considered a mechanism for the inactivation of tumor suppressor pathways in several types of cancer. These changes can impact gene expression, but how this may contribute to the process of tumorigenesis requires further investigation. 

Recent advances in the study of the role of epigenetics in the progression of osteosarcoma have increased the understanding of the pathogenesis of this disease, an area which is complex and not well defined. Altering gene expression and the signaling pathways that control the cell cycle and apoptosis can contribute to the tumorigenic process and cell transformation from a normal to a malignant phenotype. This paper serves to review what is currently known about the effects of aberrant methylation and other epigenetic mechanisms on the regulation of cell cycle and apoptosis in osteosarcoma.

## 2. Rb Pathway

**Figure 1:**
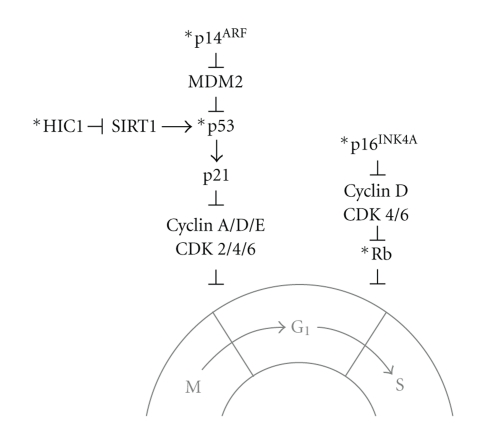
Schematic model of epigenetic events that regulate cell cycle progression in osteosarcoma. The cell cycle regulators Rb, p53, p16^INK4A^, p14^ARF^, and HIC1 have been found to be hypermethylated at the gene promoter in osteosarcoma (*). These alterations may contribute to dysregulation of cell cycle control (loss of the G1 checkpoint) and may promote tumor development.

Retinoblastoma protein (Rb) is a tumor suppressor protein that is inactivated in several types of cancer [10]. It has been shown to play a role in cell cycle control by inhibiting entry into the S-phase, thus creating a G1 checkpoint [11, 12]. About 70% of human primary osteosarcoma tumors have molecular aberrations in the Rb gene. The most common alterations include genetic deletions, mutations, and structural rearrangements [1, 13–15]. While inactivation by hypermethylation of the Rb gene has been shown to contribute to pathogenesis of other tumor types such as retinoblastoma [16], analysis of patient samples has suggested that this inactivation may not play an essential role in the progression of osteosarcoma. In one study, only 6 of 76 patients displayed heterozygous Rb methylation and 6 out of 41 patients displayed Rb promoter methylation. It is important to note, however, that loss of the Rb gene was only detectable in 37.2% of these patients, which is considerably lower than previously reported data [17]. Therefore, further analysis of Rb methylation in osteosarcoma is warranted. It has been shown that Rb-dependent G1 arrest involves p16^INK4A^ inhibition of cyclin D/cdk4 and cyclin D/cdk6 complexes, which normally initiate the phosphorylation of Rb [18]. Therefore, alterations in Rb, cyclin D, cdk4/6, or p16^INK4A^ may result in a loss of the G1 checkpoint, leading to the accumulation of genetic damage which may contribute to tumor development (Figure 1). Until recently, epigenetic modifications of the p16^INK4A^ gene, a tumor suppressor that is often altered in osteosarcoma cell lines [19], were not investigated. In a study with p16-negative osteosarcoma samples, 8/15 had total or partial CpG methylation of the p16^INK4A^ promoter and 6/15 were pRb-negative [20]. Overall, these data suggest that in addition to other mechanisms of Rb pathway inhibition, promoter methylation of either Rb or p16^INK4A^ may play a role in the disruption of cell cycle control, promoting the development of osteosarcoma.

## 3. p53 Pathway

The p53 gene (TP53) is known as the most commonly mutated gene in human cancers [1, 21–23]. P53 plays an important role in the regulation of apoptosis, cell cycle arrest, and DNA repair. When DNA damage occurs, p53 upregulates WAF/CIP resulting in increased p21 protein. P21 can then bind to and inhibit G1-S/CDK and S/CDK complexes to arrest cell division. If damage is irreparable, activated p53 can directly regulate the expression of apoptotic genes, resulting in the initiation of apoptosis [23]. The frequency of p53 alterations in osteosarcoma ranges from ~30% point mutations to ~80% allelic loss, suggesting that p53 status plays an important role in the tumorigenesis of osteosarcoma [1]. However, few groups have investigated the role of epigenetic regulation of p53 pathways in osteosarcoma. Recently a novel protein, hypermethylated in cancer (HIC1), was identified to modulate p53-dependent apoptosis (Figure 1). Inactivation of HIC1 results in the upregulation of SIRT1 deacetylase which then deacetylates and inactivates p53. This results in the circumvention of apoptosis and then cells are able to survive DNA damage, a process that may promote tumor development [24]. In order to investigate the mechanism for loss of function of HIC1 and p53 in osteosarcoma, Chen et al. analyzed the regulation of HIC1 in tumors of HIC1^+/−^ p53^+/−^ mice. Eight of 13 osteosarcomas demonstrated HIC1 1b promoter hypermethylation and 2/13 had hypermethylation of the HIC1 1a promoter. Both alterations were associated with loss of HIC1 expression, whereas tumors with abundant HIC1 expression had no apparent hypermethylation of the HIC1 promoter. In addition, 2/4 osteosarcomas in p53^+/−^ mice had abundant HIC1 1b hypermethylation. This suggests that loss of HIC1 function resulting from promoter hypermethylation, along with inactivation of p53, is associated with the development of osteosarcoma. To further examine whether HIC1 promoter hypermethylation with p53 inactivation was important in the development of human osteosarcomas, Chen et al. analyzed 44 osteosarcoma patient samples. It was demonstrated that 8/21 (38%) tumors with p53 mutations and 2/23 (9%) without p53 mutations were characterized by HIC1 promoter hypermethylation [25]. In addition, it has been found that 17% of pediatric osteosarcomas display hypermethylation of the HIC1 promoter [26]. This further validates that in addition to mutation or deletion of the p53 gene, regulation of the p53 pathway by HIC1 promoter hypermethylation resulting in p53 inactivation also plays an important role in the development of osteosarcoma. 

## 4. p14^ARF^/CDKN2A

The p14^ARF^ protein, which is encoded by the CDKN2A gene, is critical in the regulation of cell cycle control (Figure 1). P14^ARF^ regulates p53 function by inhibiting MDM2, allowing p53 to upregulate p21 expression. P21 can then bind to and inactivate cyclin/CDK complexes, resulting in G1 arrest [27]. In an examination of tissue samples from 59 osteosarcoma patients, it was demonstrated that epigenetic alterations in p14^ARF^ correlated with poor prognosis. Using methylation-specific polymerase chain reaction (PCR), it was shown that 15/32 (47%) osteosarcomas had aberrant methylation of the p14^ARF^ gene (CDKN2A) promoter. As anticipated, these 15 osteosarcomas with methylated p14^ARF^ showed negative or weak expression of the p14^ARF^ protein. This confirms that methylation of the p14^ARF^ gene promoter is associated with loss of protein expression. Methylation did not correlate with age, gender, location of tumor, tumor volume, stage, histologic subtype, or chemotherapeutic response. Interestingly, the patients with p14^ARF^ methylation had a lower median survival than the patients without p14^ARF^ methylation, which was statistically significant according to Kaplan-Meier's survival analysis. This suggests that aberrant p14^ARF^ promoter methylation correlates with poor survival in patients with osteosarcoma. In addition, 9/14 patients with p14^ARF^ promoter methylation developed metastases. Associated deaths correlated with the incidence of metastasis but were not significant due to the size of the cohort [28]. Overall, these data suggest that p14^ARF^ promoter methylation may result in the loss of p53-dependent G1 arrest, which may promote tumor development. However, further study into the significance of p14^ARF^ promoter methylation in primary osteosarcoma and metastasis is warranted and may provide further insight into the importance of p14^ARF^ methylation in osteosarcoma progression.

## 5. RASSF1A

Ras association domain family 1A (RASSF1A) is a newly identified tumor suppressor gene that is involved in pathways regulating cell cycle arrest and apoptosis. Although unclear, RASSF1A has been identified to be involved in death receptor-dependent apoptosis [29]. RASSF1A has been demonstrated to be silenced in cancers of the ovary, nasopharynx, kidney, stomach, prostate, urinary bladder, thyroid, and neuroblastoma [30–37]. The method of RASSF1A silencing in all of these tumor types was determined to be a result of aberrant methylation of the RASSF1A promoter. Recently, RASSF1 was also shown to be a tumor suppressor in osteosarcoma. Lim et al. investigated the expression of RASSF1A in primary osteosarcomas and cell lines and demonstrated a lack of RASSF1A expression in 4/10 primary and 5/6 cell lines. Upon treatment of these RASSF1A-negative cell lines with the demethylating agent 5-aza-2′-deoxycytidine, RASFF1A expression was upregulated. This suggests that RASSF1A promoter methylation may be a possible mechanism for the transcriptional silencing of RASSF1A. In contrast with these results, Lim et al. found that several primary osteosarcomas and one cell line (SAOS-2) did not display methylation of the RASSF1A promoter [38]. Therefore, further studies incorporating a greater number of osteosarcoma samples and cell lines are warranted. Additionally, Hou et al. performed a thorough analysis of promoter hypermethylation of a wide of array of genes in osteosarcoma and normal tissues using Q-MSP analysis. RASSF1A promoter hypermethylation was present in 14.29% (mean) of tumor tissues and in 1.29% (mean) of normal tissues, demonstrating that promoter methylation is a method of RASSF1A silencing in osteosarcoma [39]. This validates that loss of RASSF1A by promoter methylation may play a role in the development of osteosarcoma by dysregulation of cell cycle control and apoptosis. 

## 6. Monomethyl Histone H3 Lysine 27

Several groups have demonstrated the various histone modifications that occur during apoptosis, termed the “apoptotic histone mark” [40]. Histone tails are known to be posttranslationally modified by acetylation, methylation, phosphorylation and ubiquitination of lysine, and/or arginine residues [41, 42]. One such modification, methylation of lysine 27 in histone H3, was associated with gene repression and has been implicated in tumorigenesis [43–45]. In osteosarcoma cells, induction of lysine 27 methylation in histone H3 has been associated with caspase-dependent apoptosis and cell cycle arrest, suggesting that this epigenetic mark may play a possible role in these processes [46]. Conversely, osteosarcoma cells undergoing apoptosis displayed elevated levels of monomethylated histone H3 lysine 27 [47]. These studies are the first to provide evidence that epigenetic modification of histone H3 by lysine 27 methylation may be linked with apoptosis in osteosarcoma. Although a correlative study, this finding suggests a possible role for histone modifications in the induction of apoptosis in osteosarcoma.

## 7. Discussion

Osteosarcoma is a relatively rare disease, accounting for about 5% of all pediatric cancers. However, more than 30% of patients with osteosarcoma die of pulmonary metastasis within 5 years after diagnosis [48]. While adjuvant chemotherapy has improved overall survival rates compared to surgery alone, the fatality rates have remained unchanged for more than 20 years. Therefore, there is an ongoing need for new therapeutic strategies and a more thorough understanding of the genetic and molecular mechanisms that participate in the development of osteosarcoma and in the metastatic process, particularly to the lung. Multiple pathways have been implicated in the pathogenesis of osteosarcoma. Specifically, pathways involving cell cycle and apoptosis have been found to play a role in tumorigenesis. While there is abundant evidence that pathways involving p53, Rb, and many key mediators of cell cycle regulation and apoptosis contribute to osteosarcoma, how these critical genes are altered is not clearly understood. Recent advances in the study of epigenetics have shown that these pathways may be regulated by methylation, histone modifications, and other epigenetic mechanisms. This paper highlights the importance of these epigenetic events in the development of osteosarcoma, specifically pertaining to cell cycle regulation and apoptosis. Overall, a growing body of evidence suggests that use of therapeutic agents that target epigenetic mechanisms may be beneficial for patients with osteosarcoma.
